# Localisation rare de la tuberculose: la ténosynovite des doigts

**DOI:** 10.11604/pamj.2014.17.270.3807

**Published:** 2014-04-12

**Authors:** Kaouther Ben Abdelghani, Kaouther Maatallah, Faida Ajili, Leila Souabni, Ahmed Laatar, Leith Zakraoui

**Affiliations:** 1 Service de Rhumatologie, Hôpital Mongi Slim La Marsa, Université de Tunis El Manar, Faculté de Médecine de Tunis, Tunis, Tunisie; 2 Service de Médecine Interne, Hopital Militaire de Tunis, Université de Tunis El Manar, Faculté de Médecine de Tunis, Tunis, Tunisie

**Keywords:** Ténosynovite, tuberculose, main, tenosynovitis, tuberculosis, hand

## Abstract

La ténosynovite tuberculeuse est une localisation rare de la tuberculose. Le diagnostic en est souvent tardif en raison de manifestations cliniques souvent pauvres et chroniques. Nous rapportons une observation de ténosynovite tuberculeuse du 2ème rayon de la main droite d’évolution favorable sous traitement antituberculeux.

## Introduction

Bien que rare dans les pays développés, la tuberculose est une des maladies les plus redoutées dans les pays sous-développés et en voie de développement où elle constitue un véritable problème de santé publique. Ses présentations inhabituelles tel que la ténosynovite tuberculeuse sont souvent méconnues et s'associent à un retard diagnostique et thérapeutique d'autant plus que la preuve histologique formelle peut parfois manquer. Nous en rapportons une nouvelle observation de ténosynovite tuberculeuse dont le diagnostic a été retenu sur des éléments de présomption.

## Patient et observation

**Figure 1:**
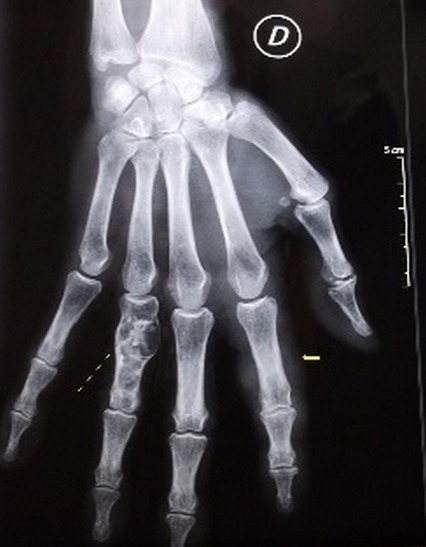
Radiographie standard de la main droite: épaississement des partie molles du 2ème rayon droit+ une lésion diaphysaire de la 4ème IPP droite, lytique et condensante, cernée d'un fin liseré d'ostéosclérose avec une corticale soufflée compatible avec un chondrome

**Figure 2:**
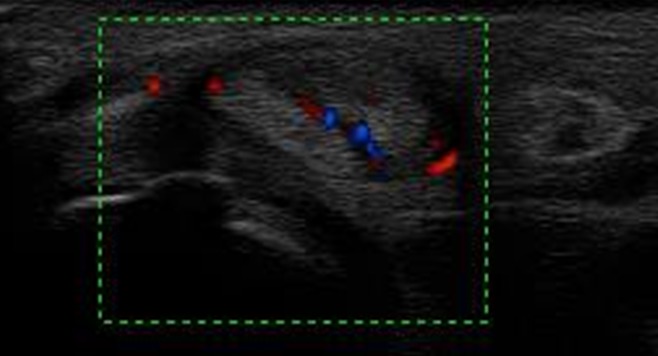
Coupe transversale du tendon fléchisseur du 2ème rayon droit: épanchement liquidien dans la gaine tendineuse des tendons fléchisseurs superficiel et profond du 2ème rayon droit, hyper vascularisée au doppler puissance

**Figure 3:**
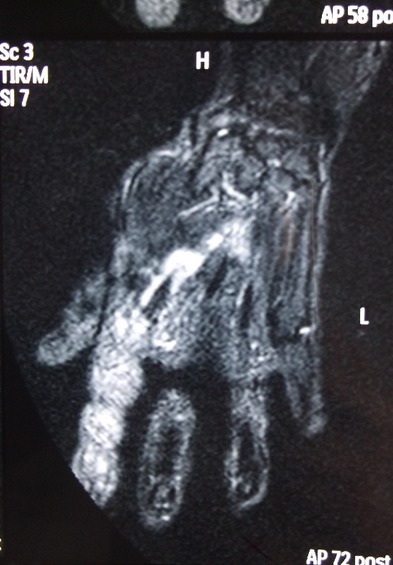
Coupe sagittale de la main droite IRM T1 après injection de gadolinium: Prise de contraste de la gaine synoviale

**Figure 4:**
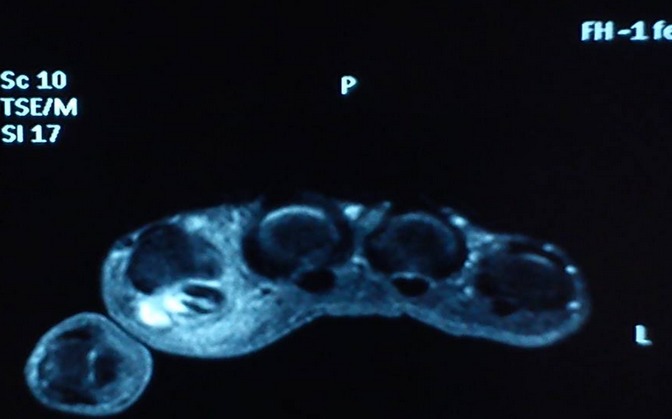
Coupe axiale de la main droite IRM T1 Gadolinium: Épaississement de la gaine synoviale avec prise de contraste après Gadolinium

Mme T.Z âgée de 77 ans sans antécédents pathologiques notables a consulté pour une tuméfaction douloureuse du deuxième rayon de la main droite évoluant depuis quatre mois avec depuis trois jours apparition de signes inflammatoires locaux. Elle n'a pas présenté par ailleurs aucune autre plainte articulaire ni notion de fièvre ou altération de l’état général. Il n'y avait pas de notion de traumatisme. L'examen a objectivé une ténosynovite du fléchisseur du 2ème doigt droit. Le reste de l'examen ostéo-articulaire, pleuropulmonaire et général était sans particularité. La biologie a révélé un syndrome inflammatoire avec une VS à 40 sans hyperleucocytose. L'intradermo-réaction à la tuberculine était positive à 15mm. La radiographie des mains a montré un épaississement des parties molles du 2ème rayon droit. Il existait par ailleurs une lésion diaphysaire de la 4ème IPP droite, lytique et condensante, cernée d'un fin liseré d'ostéosclérose avec une corticale soufflée compatible avec un chondrome (Figure 1). Une échographie articulaire a montré un épaississement tissulaire associé à un épanchement liquidien siégeant dans la gaine tendineuse des tendons fléchisseurs superficiel et profond du 2ème rayon droit, hyper vascularisée au doppler puissance (Figure 2). Une IRM des mains a été alors pratiquée et a objectivé un épaississement de la gaine synoviale des tendons fléchisseurs superficiel et profond du 2ème rayon droit se rehaussant après injection de Gadolinium. Il s'y associait une collection liquidienne en hypo signal T1 et hyper signal hétérogène en T2 autour du tendon (Figure 3, Figure 4). Par ailleurs, le chondrome du 4ème rayon a été également visualisé. L'indication d'une biopsie chirurgicale a été alors posée et l'examen anatomo-pathologique a révélé un granulome gigantocellulaire mais sans nécrose caséeuse. La recherche de BAAR à partir des fragments prélevés était négative et la culture sur les milieux de Löwenstein-Jensen et Colet était stérile. La radiographie de thorax était sans anomalies ainsi que la recherche de Bacille de koch (BK) dans les crachats et les urines. Devant l’évolution insidieuse, le résultat de l'intradermo-réaction, les données de l'examen anatomo-pathologique et en raison du caractère endémique de la tuberculose dans notre pays, le diagnostic de ténosynovite tuberculeuse a été le plus probable. Ainsi, la patiente a été traitée par isoniazide(INH), rifampicine(RIF), pyrazinamide (PZA) et ethambutol (EMB) pendant deux mois puis par INH et RIF pendant quatre mois. Devant l'importance des nausées et de l'anorexie secondaires à la prise des antituberculeux la patiente a tenue à arrêter le traitement au bout de 6 mois L’évolution a été marquée par une disparition complète de la tuméfaction dès le deuxième mois du traitement avec un recul actuel d'un an.

## Discussion

L'atteinte de l'appareil locomoteur représente 1 à 5% des manifestations extra pulmonaires de la tuberculose. La ténosynovite tuberculeuse présente 5% des tuberculoses ostéo-articulaires et elle prédomine au poignet et à la face palmaire de la main [1, 2], l'atteinte des fléchisseurs reste beaucoup plus fréquente que les extenseurs. Le clinicien évoque rarement le diagnostic de tuberculose face à un tableau de ténosynovite chronique et le délai diagnostique est souvent long. La ténosynovite tuberculeuse est marquée par un début insidieux. La tuméfaction devient évidente après plusieurs mois, la mobilité devient limitée et elle peut évoluer vers la fistulisation. Les signes inflammatoires locaux sont discrets. Notre patiente n'a consulté qu'après quatre mois du début d’évolution de la maladie ce qui témoigne de l’évolution insidieuse de la symptomatologie. Un syndrome du canal carpien peut s′observer en cas d′atteinte des fléchisseurs au poignet [2]. Le tableau clinique peut également simuler une ténosynovite de De Quervain [3, 4]. L'inoculation peut être directe ou par dissémination hématogène à partir d′un foyer à distance. Il faut systématiquement rechercher d'autres foyers tuberculeux, essentiellement pleuropulmonaire mais aussi osseux, ganglionnaires, rénaux. Chez notre patiente, la ténosynovite était primitive puisqu'aucune autre infection ni contiguë ni à distance n'a été identifiée. Cette infection peut être favorisée par un traumatisme dans 30% des cas [2], un travail forcé, une infiltration de corticoïdes, une immunodépression (infection par le VIH et corticothérapie au long cours), l'alcoolisme ou un âge >60 ans comme dans le cas de notre patiente [1, 5].

Biologiquement, il existe souvent un syndrome inflammatoire, la vitesse de sédimentation (VS) est un élément très évocateur mais non spécifique, elle est en règle supérieure à 60 mm. L’échographie est un examen utile pour confirmer le diagnostic de ténosynovite et montrer son extension comme dans le cas de notre patiente. Elle permet d′objectiver une augmentation du volume de la gaine synoviale, formant un manchon autour du tendon. On peut observer un épaississement tendineux ou une collection liquidienne abcédée [4]. L’échographie est également utile pour la réalisation d'une ponction écho-guidée avec une étude PCR du liquide prélevé évitant ainsi le recours à la chirurgie [6]. L′IRM est certainement l'examen le plus utile et le plus sensible, la synoviale granulomateuse des gaines tendineuses est typiquement de signal intermédiaire en T1, rehaussé par l'injection de gadolinium et en hyper signal en T2. L’épanchement est en hypo signal T1 et hyper signal T2. L'IRM montre la prolifération synoviale mais aussi, dans certains cas, la formation d'abcès et la destruction des os adjacents, qui étaient absents chez notre patiente [7]. La preuve bactériologique n'est présente lors de l'examen direct que dans 20% des cas et les cultures sont négatives dans 35 à 45% des cas [8] Les méthodes d′amplification génique à partir du liquide synovial sont plus sensibles et permettent une détection rapide et spécifique du BK [1]. L'absence de BK aux cultures ne peut et ne doit en rien infirmer le diagnostic. Concernant l’étude histologique, Kanavel et al [9] avait décrit trois stades évolutifs: le 1er stade correspondant à un exsudat séreux, le second à un tissu de granulation avec ou sans l′aspect en « grain de riz » (Le cas de notre patiente) et le 3ème stade, qui est plus tardif, correspond à des fongosités associée à une nécrose extensive, caséeuse. Chez notre patiente et en dépit de l'absence de la nécrose caséeuse, l'origine tuberculeuse a été retenue devant un faisceau d'arguments épidémiologiques, cliniques, biologiques et anatomo-pathologiques. Le diagnostic différentiel des ténosynovites tuberculeuses peut se faire avec une infection aux autres mycobactéries responsables d'une ténosynovite granulomateuse: Une mycose, une brucellose, une ténosynovite sur corps étranger ou une sarcoïdose [1, 2]. Chez notre patiente, la sarcoïdose était peu probable en raison de l'absence de l'anergie à la tuberculine. La synovite à piquant a été également évoquée mais il n'y avait pas de notion de traumatisme à l'interrogatoire. Une fois le diagnostic établi, le traitement est très simple et le patient guérit en général avec peu ou pas de séquelles. Le traitement repose sur une quadrithérapie initiale associant INH, RIF, PZA et EMB pendant 2 mois. Au bout de 2 mois et en l'absence de résistance, le traitement devient une bithérapie associant INH et RIF. La durée minimale du traitement antituberculeux au cours des tuberculoses ostéo-articulaires reste à l'heure actuelle mal codifiée.

D'après les recommandations américaines les plus récentes, les traitements de la tuberculose ostéo-articulaire comportant de la RIF et ayant une durée de 6 à 9 mois étaient aussi efficaces que des traitements ne comportant pas de la RIF et ayant une durée de 18 mois [10]. Chez notre patiente la durée du traitement était de seulement 6 mois mais l’évolution était très favorable avec un recul d'un an. Le recours à la chirurgie n′est envisagé que pour la biopsie, le drainage de lésions nécrosées étendues ou en cas de compression nerveuse.

## Conclusion

Les infections tuberculeuses de la main, bien que rares, sont encore régulièrement rencontrées. L’évolution est insidieuse et le diagnostic reste difficile au stade de début. Les examens biologiques, l’échographie et l'IRM permettent d'orienter le diagnostic. Le diagnostic de certitude est fourni par l’étude histologique. Le traitement repose sur la chimiothérapie antituberculeuse, le traitement chirurgical est devenu beaucoup plus rare, devant être discuté au cas par cas.
